# Ciguatera Toxin Syndrome from Amberjack Ingestion as a Cause of Chronic Dermatitis with Episodic Erythema

**DOI:** 10.7759/cureus.46755

**Published:** 2023-10-09

**Authors:** Matthew D Lucas, Mahlon R Kile, Malika P Ganguli, Ves Dimov

**Affiliations:** 1 Internal Medicine, American University of the Caribbean School of Medicine, Cupecoy, SXM; 2 Internal Medicine, Ross University School of Medicine, Bridgetown, BRB; 3 Family Medicine, Ross University School of Medicine, Bridgetown, BRB; 4 Allergy and Immunology, Cleveland Clinic Hospital of Florida, Weston, USA

**Keywords:** ciguatera toxicity, ciguatera fish poisoning, ciguatera toxin syndrome, skin rash, dermatitis, reef fish, ctx, ciguatera, amberjack

## Abstract

Bioaccumulation of naturally produced ciguatoxin (CTX), such as that in ciguatera poisoning, continues to be a subject of great interest. In this condition, CTX is ingested by subtropical and tropical reef fish. Humans consume the fish species, and CTX is absorbed through the gastrointestinal tract and binds voltage-gated sodium channels on nerve terminals to cause neurological, gastrointestinal, cardiac, and rare dermatological clinical manifestations. In this present case, we discuss a 65-year-old female who presented with acute loose bowel movements and generalized pruritus of her anterior chest wall, abdomen, and bilateral upper and lower extremities 48 hours after consumption of amberjack fish. The patient was treated with intravenous corticosteroids and epinephrine and discharged with an oral corticosteroid taper. After appropriate treatment protocol, the patient continued to have pruritus with a burning sensation in her extremities with a rare skin dermatitis. Subsequent treatment included topical corticosteroids and moisturizing lotion to create a skin barrier, fexofenadine for pruritus control, and gabapentin and amitriptyline for paresthesia. This case demonstrates the need for continued research and patient education into the broad clinical manifestations that present as life-altering ciguatera poisoning.

## Introduction

Ciguatoxins (CTX) are naturally occurring toxins of algal origin found in subtropical regions. These toxins bioaccumulate in the food chain, especially within reef fish. According to the Center for Disease Control and Prevention [1], these reef fish include barracuda, grouper, red snapper, and amberjack. This marine toxin originates with molecular precursors, such as gambiertoxin-4A produced by marine dinoflagellates [2]. One example of these dinoflagellates includes Gambierdiscus toxicus. Certain Herbivorous fish consume Gambierdiscus toxicus and other dinoflagellates with CTX precursors and via biotransformation and acid-catalyzed spiro-isomerization of precursors within a hepatic metabolic pathway within the fish, CTX is formed. Carnivorous fish, including reef fish, then consume the herbivorous fish, which causes the carnivorous species to ingest CTX, bioaccumulating further up the food chain [2]. Subsequently, humans are exposed to CTX upon consuming these carnivorous fish. CTX is absorbed through the gastrointestinal tract into the bloodstream. This leads to ciguatera fish poisoning (CFP), caused by the binding of CTX to voltage-gated sodium channels, allowing for frequent depolarization and a neuroexcitatory state [3]. 

The incidence of CTX ingestion is estimated to be about 50,000 cases per year globally [3]. It can cause gastrointestinal, cardiovascular, neurologic, and dermatological symptoms. Interestingly, due to variations in CTX biomolecular organization, particular symptomatology seems to be distributed based on geographical region, ranging from the Caribbean Sea to the Pacific and Indian Oceans [4]. In the Caribbean, gastrointestinal symptoms are predominant, followed by peripheral neurologic symptoms to a lesser extent. The predominant gastrointestinal symptoms include nausea, vomiting, diarrhea, and abdominal pain lasting 24-72 hours [5]. In the Pacific and Indian Ocean regions, neurologic symptoms prevail. CTX-induced neurologic symptoms manifest in a wide variety of ways, including paraesthesia of the perioral region and extremities, abnormal temperature-related sensations, metallic taste, pruritus, arthralgia, lower extremity myalgias, sensation of loss of teeth, non-specific headaches, hyporeflexia, and dysphagia. Severe toxicity may result in coma, respiratory depression, and death [4]. Neurobehavioral signs, such as fatigue, anxiety, and depression, can also present in CFP patients. These neurological clinical signs are chronic in nature and last one week to multiple years. In contrast, those patients presenting with cardiovascular symptoms of CFP, including hypotension and bradycardia, often see resolution of symptoms within 48-72 hours [4]. In this report, we present a case of CFP with symptoms of dermatitis and unique erythema, an interesting and otherwise unreported manifestation.

## Case presentation

This is a case of a 65-year-old female who presented to the allergy clinic. Her past medical history is notable for chronic rhinitis, allergic rhinitis, and two isolated events of hand numbness while playing tennis. Approximately six weeks prior to the visit to our allergy clinic, she visited the emergency department following loose bowel movements and generalized pruritus of her anterior chest wall, abdomen, and bilateral upper and lower extremities 48 hours after amberjack fish ingestion. She was given intravenous corticosteroids and epinephrine and discharged with an oral corticosteroid taper. However, she continued to have pruritus with a burning sensation in her extremities with an erythematous rash. Although initially treated for a suspected allergic reaction, her symptoms were more suggestive of a non-IgE mediated system due to the delayed 48-hour symptoms. Furthermore, the presence of non-IgE-mediated symptoms dismissed a possible diagnosis of adult-onset atopic dermatitis and contact dermatitis. Thus, we concluded that her reactions are ciguatera toxin-mediated.

Pruritus labs completed did not show a cause for her symptoms. Her neurologic exam was notable for decreased ankle reflexes rated one out of four bilaterally; however, otherwise, within normal limits for muscle tone, bulk, strength, pinprick, and light touch. She was treated with multiple topical corticosteroids, moisturizing lotion to create a skin barrier, and fexofenadine for pruritus control. Paresthesias were moderately relieved with gabapentin and amitriptyline. Unfortunately, this patient’s symptoms have not completely resolved, and she still experiences episodic erythema and pruritus of her bilateral hands.

## Discussion

The pathogenesis of CFP involves the absorption of CTX into the bloodstream via the gastrointestinal tract. According to Nicholson et al., CTX binds to site 5 of the alpha subunit with high specificity and affinity to voltage-gated sodium channels, which are heavily distributed among excitatory cells. This subsequently causes a decreased threshold to open the voltage-gated sodium channel, increasing sodium influx [6]. This results in more frequent and premature depolarization, which increases neuroexcitation. This elevated activity manifests as a broad range of symptoms.

While documented cases of CFP in current literature describe an extensive manifestation of symptoms, including neurological, gastrointestinal, and cardiac, patients presenting with a dermatitis rash and episodic erythema are not discussed to our knowledge. The broad range of symptoms causes numerous cases of CFP poisoning to be misdiagnosed, leading to poor patient outcomes. Due to the rare presentation in the presented case, it is apparent that the vast symptomatology that presents as CFP is a frontier that must be explored. Further exploration will inform future clinicians of this harmful condition and determine therapeutic approaches to reduce acute and chronic symptoms.

Although our patient's presentation may suggest a clinical manifestation of allergy-induced sensitization, some factors must be considered that imply otherwise. This patient's onset of symptoms occurring 48 hours after consumption of ciguatoxin-containing fish alludes to a clinical picture of toxin exposure and response. In contrast, an IgE-mediated allergic response would occur soon after ingestion of CTX. Our patient’s burning sensation in her hands and feet is more consistent with toxin-mediated nerve damage and nerve-related pain, especially in relief provided with gabapentin and amitriptyline. It is typical for neurological symptoms of CFP to linger on. A case presented by Winters [7] showed ciguatoxin presenting with persistent neurological deficits and improvement of symptoms with similar medications. Along with this presentation is generalized pruritus, a known neurologic symptom that presents and lasts like the others. Similarly, a case described by Bailey et al. [8] showed CFP presenting as pruritus of the extremities in two medical students who consumed parrotfish during an elective rotation in the Cook Islands. Their symptoms fluctuated with time but resolved in 10 weeks with treatment of antihistamines.

The manifestation of episodic erythema is the most interesting about this patient's presentation. To our knowledge, no literature in the English language has documented a case of episodic erythema of the hands and feet induced by CFP. 

Touska et al. [9] described a study in which calcitonin gene-related peptide (CGRP) is related to atopic dermatitis. Further examination demonstrated that pacific ciguatoxin-1 (P-CTX-1) induced the potent dose-dependent release of CGRP from nerve terminal in mouse and rat skin preparations detected by analyzing the quantity of immunoreactive CGRP by Enzyme-Linked Immunoassay (ELISA). This made the mouse skin thinner and exposed more nerve endings than the rat skin, which is thicker.

Although the research performed by Touska et al. relates to that of rodents, this could propose a possible cause for the atypical clinical dermatitis and erythema manifestation of CFP in the presented case. Although we cannot make a definitive conclusion as to why our patient experienced this erythema with Touska’s research, it does provide insight into possible future management via understanding the case-by-case sensitivities of patients. The CGRP test kit revealed that CTX induces the release of CGRP from nerve terminals by activating specific voltage-gated sodium channels. In contrast, the test kit revealed lidocaine, tetrodotoxin (TTX), and the activation of voltage-gated calcium channels reduced the release of CGRP. These findings may support future innovative therapeutics that may serve to reduce CTX levels and clinical manifestations seen in CFP patients. 

As previously stated, wide symptomatology manifestations have been reported in the current literature. Winter reported a case in which Ciguatera poisoning manifested as nausea, vomiting, and diarrhea, with up to 10 bowel movements per day after a couple consumed local fish on a trip in Costa Rica. Neurological symptoms of tingling and numbness exacerbated by air movement over the extremities, cold allodynia, and photophobia followed the previous gastrointestinal symptoms. Their gastrointestinal symptoms lasted one week, while their neurological symptoms lasted three months. No rash was noted on the physical exam. A clinical diagnosis of CFP was established, and amitriptyline was initiated. Amitriptyline therapy was initiated for three months, and minimal neurological symptoms persisted.

In contrast, the Canada Communicable Disease Report [10] described a 65-year-old male presenting with diarrhea and shivering after consuming barracuda. Interestingly, a rash was noted on the physical exam. The rash was located on his hands, feet, and genitalia. He was treated with loperamide, which caused the diarrhea to subside. Interestingly, he noticed an increase in symptoms after consumption of wine with dinner. The patient refrained from alcohol and fish consumption, and symptoms diminished.

Interestingly, based on geographical distributions, different CTX subclassifications have been identified. These geographical distributions include the Pacific Ocean (P-CTX-1), Indian Ocean (I-CTX), and Caribbean Sea (C-CTX-1) [5]. While ciguatoxins are lipid-soluble polyethers, different biochemical structures have been isolated for P-CTX-1 and C-CTX-1, but not I-CTX. According to Nicholson et al., the level of toxicity varies between isolated subtypes, but P-CTX-1 isolated structures are noted to be the most toxic in animal models. Still, this information does not clearly answer why our patient presented with episodic erythema of her hands.

These cases highlight the diverse symptomatology related to CFP, whether gastrointestinal, neurological, cardiac, or dermatological. Accordingly, the present case serves as another example of the diversity of presentations within the spectrum of diagnoses, with the rare dermatological symptoms of dermatitis and episodic erythema being the most noteworthy. This case reinforces not only the idea that cases of ciguatoxin exposure can be highly variable but perhaps there is an element of both ciguatoxin exposure and allergy-related symptoms that may appear, as in the presented case. As clinicians, we are responsible for completing a comprehensive history and remaining vigilant, using all resources and information to decipher the correct diagnosis. Education regarding avoiding exposure to ciguatoxin is paramount. Treatment of ciguatera fish poisoning is in its infancy. Intravenous (IV) fluids and epinephrine are used acutely. Topical corticosteroids and fexofenadine are used for symptomatic improvement. Gabapentin and other Tricyclic antidepressants like amitriptyline are used for pain/neuropathy. Unfortunately, minimal work has been done to explore the treatment and efficacy of said treatment in the other vast host of symptoms. Future investigations should include the efficacy of treatment options for patients experiencing the vast symptoms that may occur.

## Conclusions

This case emphasizes the importance of recognizing and understanding rare instances like dermatitis and erythema in ciguatera poisoning in allergy and immunology. Distinctive clinical manifestations, such as the present case, call for a continued dedication to acquiring a detailed and comprehensive patient history and an awareness of ciguatera poisoning, ultimately leading to improved diagnostic rates and management. To improve the quality of life of these patients, this case dictates that future management of these patients should involve proper education among patients in high-risk geographical regions for preventive care rather than waiting to treat the patient’s symptoms. In the event of such poisoning, multiple cases report mannitol, intravenous fluids, corticosteroids, gabapentin, or amitriptyline protocols being utilized as the first-line treatment for Ciguatera poisoning. Future diagnostic measures may highlight the benefit of enzyme-linked immunosorbent assay (ELISA) calcitonin gene-related peptide (CGRP) test kits for detection as a sensitive and specific marker of elevated CTX in a patient with new-onset gastrointestinal, neurological, or dermatological manifestations. Likewise, the CGRP test kits have revealed potential therapeutic approaches by evaluating inhibitors of CGRP release in mouse and rat models. These inhibitors of CGRP release, such as TTX and lidocaine, may serve as rescue treatments in patients with CFP. This case and the previous ones serve as an awareness of the diagnosis and the variety of important yet overlooked manifestations while highlighting treatment and prevention options for patients with this captivating condition.
